# Time-Efficient Convolutional Neural Network-Assisted Brillouin Optical Frequency Domain Analysis

**DOI:** 10.3390/s21082724

**Published:** 2021-04-13

**Authors:** Christos Karapanagiotis, Aleksander Wosniok, Konstantin Hicke, Katerina Krebber

**Affiliations:** Bundesanstalt für Materialforschung und-Prüfung, Unter den Eichen 87, 12205 Berlin, Germany; Aleksander.Wosniok@bam.de (A.W.); Konstantin.Hicke@bam.de (K.H.); Katerina.Krebber@bam.de (K.K.)

**Keywords:** distributed Brillouin sensing, convolutional neural networks, Brillouin optical frequency domain analysis, distributed fiber-optic sensors, temperature and strain sensing

## Abstract

To our knowledge, this is the first report on a machine-learning-assisted Brillouin optical frequency domain analysis (BOFDA) for time-efficient temperature measurements. We propose a convolutional neural network (CNN)-based signal post-processing method that, compared to the conventional Lorentzian curve fitting approach, facilitates temperature extraction. Due to its robustness against noise, it can enhance the performance of the system. The CNN-assisted BOFDA is expected to shorten the measurement time by more than nine times and open the way for applications, where faster monitoring is essential.

## 1. Introduction

Brillouin distributed sensing provides spatially resolved temperature and strain information over a km-long measurement range [1]. Its area of application ranges from structural health monitoring of bridges [2], dikes [3] and pipelines [4] to condition monitoring of high voltage cables [5]. Brillouin optical time-domain analysis (BOTDA) [6], as well as Brillouin optical frequency domain analysis (BOFDA) [7], are among the techniques that have been studied extensively and can provide measurements over 100 km [8,9]. 

BOFDA makes use of amplitude modulated continuous pump waves by exploiting the time-reversal property of the fast Fourier transformations and, owing to narrow measurement bandwidth, can provide a high signal-to-noise ratio (SNR) [10]. Furthermore, since no fast electronics are needed for data acquisition [10], BOFDA is a cost-effective solution. However, due to the long measurement time that results from narrow bandwidth filtering, BOFDA is not quite attractive for long-distance sensing. 

It has been shown that machine learning can provide solutions to many problems related to and enhancing the performance of the distributed fiber optic sensors [11]. Particularly in BOTDA sensing, machine learning algorithms based on artificial neural networks (ANN) [12,13,14] and support vector machines (SVM) [15] were implemented to extract the Brillouin frequency shift (BFS) outperforming conventional algorithms based on Lorentzian curve fitting (LCF). Because the extraction of temperature or strain necessitates the estimation of the temperature or strain coefficient, respectively, machine learning models were trained to predict the measurand of interest directly from the Brillouin gain spectrum providing a more compact solution [16,17,18]. Additionally, convolutional neural networks (CNNs) were trained for denoising of Brillouin gain spectra (BGS), facilitating the estimation of the Brillouin frequency shift [19]. Furthermore, ANNs were used in BOTDA for simultaneous strain and temperature measurements [20,21]. 

In this paper, we propose, to our knowledge for the first time, a signal post-processing analysis method based on machine learning for fast and direct evaluation of temperature in BOFDA sensing. The goal of our study is to render BOFDA sensing more attractive for long-distance sensing by decreasing the required measurement time and to open the way for new applications that require faster monitoring. In BOFDA, faster monitoring, considering a fixed measurement length and spatial resolution, is feasible by reducing the amount of averaging or the Brillouin frequency scanning step and range and by broadening the bandwidth filtering. However, all this comes at the cost of noise and lower accuracy. In this study, we have trained CNNs specially designed for the evaluation of BOFDA spectra with regard to temperature. We show that this approach is more robust against noise in comparison with the conventional approach. Specifically, the CNN-assisted BOFDA can shorten the measurement time by more than nine times, paving the way towards a time-efficient ultra-long-distance BOFDA sensing.

## 2. Methods

### 2.1. Experimental Setup

**Figure 1:**
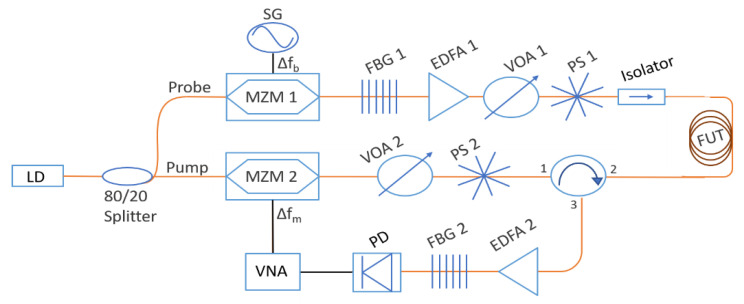
Brillouin optical frequency domain analysis (BOFDA) experimental setup. LD: laser diode; MZM: Mach–Zehnder modulator; SG: signal generator; FBG: fiber Bragg grating; EDFA: erbium-doped fiber amplifier; VOA: variable optical attenuator; PS: polarization scrambler; FUT: fiber under test; PD: photodiode; VNA: vector network analyzer.

The experimental setup is shown in Figure 1. A distributed feedback laser (DFB) provides an output optical power of 0.1 W at 1550 nm, which is split into a pump and probe path via a 20/80 polarization-maintaining optical coupler. The upper branch (probe) is responsible for the BFS tuning, while the lower branch (pump) is used for the acquisition of spatially resolved information. A Mach–Zehnder modulator (MZM 1) driven by an RF signal generator (SG) is employed to generate two sidebands and suppress the carrier (suppressed-carried double sideband [22]). The following fiber Bragg grating (FBG 1) is utilized to filter out the lower sideband. Additionally, due to the decrease in the power, an erbium-doped fiber amplifier (EDFA 1) is used. The probe branch and the pump branch include a variable optical attenuator (VOA) to adjust the optical power and a fiber squeezer-based polarization scrambler (PS) that operates at 700 kHz to reduce the polarization fading. The isolator mainly protects the components in the probe branch from the transmitted pump signal. 

In the pump branch, a second MZM (MZM 2) is employed in the linear range of the transfer function and gradually modulates the amplitude of the continuous wave in a range of modulation frequencies that are adjusted by a vector network analyzer (VNA). The Brillouin backscattered signal in the fiber under test (FUT) is amplified by a second EDFA (EDFA 2) and then passes through an FBG to filter out the Rayleigh component of the measured signal. In the end, the VNA measures the system’s response using the electrically transformed signal that is acquired by a photodiode (PD).

The measurement length *L_max_* and the spatial resolution Δ*z* are determined by the modulation frequency to step Δ*f_m_* and the maximum modulation frequency $f_{m}^{max}$ , respectively.

(1) $$\Delta z=\frac{c}{2n}\frac{1}{f_{m}^{max}-f_{m}^{min}}$$

(2) $$L_{max}=\frac{c}{2n}\frac{1}{\Delta f_{m}}$$

In this study, we measure 30 km of a standard optical fiber with a spatial resolution of Δ*z* = 25 m. To this end, according to (1) and (2) [7], Δ*f_m_* and the $f_{m}^{max}$ are set to 3 kHz and 4 MHz, respectively. We note that *c* is the speed of light in vacuum, *n* the refractive index of the material, and $f_{m}^{min}$ the minimum modulation frequency, which in our case, is equal to Δ*f_m_*. In order to conduct fast measurement, we set in the VNA only three averages and a bandwidth of 100 Hz. Moreover, in the probe branch, the Brillouin frequency sweep is performed in the range of 10.78 GHz to 10.88 GHz with a step of 20 MHz. These settings result in a total measurement time of 4 min.

We also note that the system should be linear [7,10], and thus we set a low value of pump and probe power through the VOAs. Specifically, the probe and pump powers are 27 μW and 280 μW, respectively.

### 2.2. Signal Processing

In contrast to BOTDA, where one can acquire the impulse response directly in the time domain, in BOFDA the measured quantity is the complex transfer function that can be transferred to the time domain via inverse fast Fourier transformation (iFFT). A common procedure in BOFDA signal processing before the IFFT is the application of a window function [10]. This study uses a Kaiser window [23] with a beta factor equal to 5. After windowing, the iFFT can be calculated, providing spatially resolved information. 

In this study, before the iFFT, we applied zero padding to the data to increase the Nyquist frequency 16 times. This results in 16 (instead of one) equally spaced Brillouin gain spectra (BGS) within the defined physical spatial resolution. In this way, even though the physical spatial resolution is not affected, the spatial accuracy increases [24]. 

### 2.3. Conventional & CNN-Based Approach

**Figure 2:**
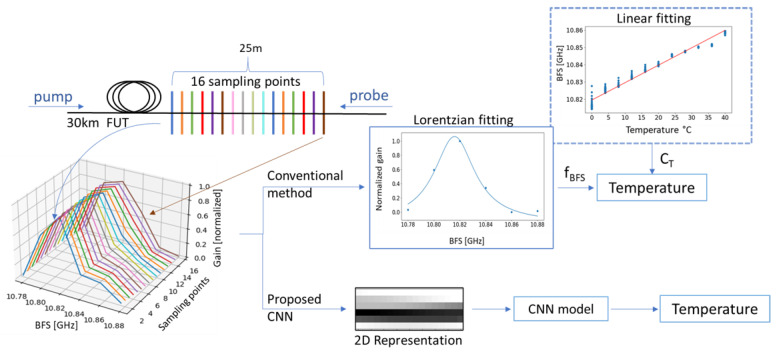
Schematic representation of the conventional and convolutional neural network (CNN)-based approach.

We propose a CNN-based approach, and we compare its performance with that of a conventional LCF-based method. A graph that provides an illustrative comparison of the two methods is shown in Figure 2. With the conventional method, one has to perform LCF to every single BGS in order to extract the BFS. In this work, we employed the Levenberg–Marquardt algorithm to perform LCF. The initial parameters for the LCF can affect the accuracy of the BFS, and in cases, with a very low SNR (usually in long fibers), their estimation can be cumbersome. The extraction of temperature is done using the Brillouin temperature coefficient C_T_, which is unique for every fiber. The BFS is expected to be a linear function of temperature, und thus the C_T_ can be estimated by a linear fitting [7], as depicted in Figure 2. Furthermore, the temperatures extracted from every 16 BGS (within the defined spatial resolution) are averaged. 

CNNs extract the most important features using their convolutional kernels, and therefore, no feature extraction is required before the training or the prediction [25]. As a result, our proposed CNN-based method requires neither an LCF to estimate the BFS nor a preliminary study of the C_T_. Thus, one can clearly determine the temperature directly from the BOFDA spectrum. In Figure 2, we show that the BOFDA spectrum entering the CNN consists of 16 BGS within the defined spatial resolution, and its output is a single value of temperature. Since the inputs of CNNs are usually images, we show a 2D representation of the BOFDA spectrum as well. 

**Figure 3:**
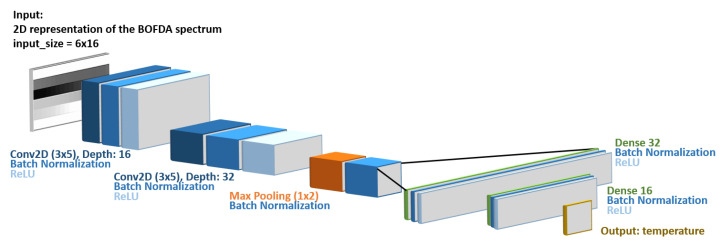
CNN architecture. The input is a 6 × 16 (Brillouin frequency steps × number of BGS) BOFDA spectrum and the output is a single value for temperature. Convolutional layers use 3 × 5 filters with a depth of 16 and 32 for the first and second layers, respectively. After flattening, two fully connected layers with 32 and 16 nodes are utilized before the output layer. Batch normalization and a ReLU activation function follow every convolutional and fully connected layer.

We designed a CNN architecture similar to one commonly used for image recognition but smaller in order to adjust to the dimensions of our images. The input consists of 6 (Brillouin frequency steps) × 16 (number of BGS) = 96 pixels, and the output is a single value giving the temperature. Similar to the VGG16 architecture [26] that is used for image recognition, our network starts with two two-dimensional convolutional layers followed by a max-pooling layer. The number of filters (depth) in the first and second convolutional layer is 16 and 32, respectively, and the filter size in both layers is (3 × 5). This asymmetric filter size performs better than the common square-shaped kernels, which is most likely due to the input’s asymmetric dimension. The same also applies to the downsampling pooling layer, which works in one direction. Then, the pooled feature map is flattened, and two fully connected layers are introduced before the output. After every layer, a ReLU activation function was used for the nonlinear mapping. To avoid overfitting [27] and to reduce the internal covariate shift problem [28], batch normalization was used. The CNN architecture is shown in Figure 3.

Additional hyperparameters that are related to the training algorithm can also influence the training process considerably [29]. In our case, we used the Adam (Adaptive Moment Estimation) [30] optimization algorithm to update the weights of the network by minimizing the error (loss function) between the labels (temperatures) and the predictions. The exponential decay rate of the 1st (β_1_) and 2nd (β_2_) moment estimates were set to 0.9 and 0.999, respectively, while the learning rate (lr) was set to 0.001. Furthermore, 125 learning epochs were found to be sufficient, and a batch size of 64 could ensure a smooth and efficient training process. 

The CNNs were implemented in TensorFlow (v. 2.0.0) [31] using the Keras library (v. 2.3.1) [32]. An NVIDIA GeForce RTX 2080 Ti 11GB RAM GPU was used for training and prediction.

### 2.4. Data Collection & Training Process

The CNN model was trained and evaluated with real experimental data that were collected using the setup and parameters reported in Section 2.1. In contrast to synthetic data, where artificial white Gaussian noise is added to ideal BGS in order to increase the generalizability of the model [13,14], the experimental data contains the actual noise that arises from the optical components [19]. The data were collected from measurements under controlled temperature conditions using a temperature chamber. 

**Figure 4:**
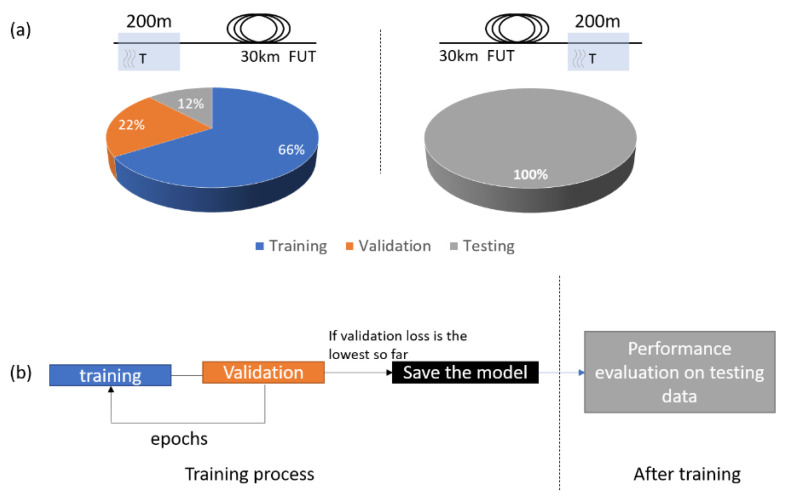
Schematic representation of the data distribution (**a**) and training pipeline (**b**).

We collected data (around 1200 images) from a 200-m strain-free segment at the beginning of a 30-km optical fiber in the temperature range from 0 °C to 40 °C with 4 °C steps. Most of the data are used for training and validation (66% and 22%, respectively), and a 12% for testing the ability of the network to generalize the unseen data. In order to verify the robustness of our method, we collected additional data (around 300 images) from another 200-m segment at the end of the 30-km optical fiber, where the SNR is reduced significantly. These data are used solely for testing. In Figure 4a, we show a sketch of the distribution of the data. 

In image recognition tasks, data augmentation is often used to help the network generalize better and avoid overfitting [33]. In our case, we applied data augmentation and specifically made use of the horizontal flipping method. Data augmentation was applied to every image of the training dataset.

In Figure 4b, we show a schematic representation of the training pipeline. After every epoch (a pass through the whole training set) the model is validated on data that are strictly not included in the training set, and if the validation loss is the lowest until that point, the model is saved or overwritten. The stored model at the end of the training phase is the one with the lowest validation loss. This training process estimates the accuracy of the model after every epoch and can determine the model that generalizes best, avoiding overfitting. 

## 3. Results

**Figure 5:**
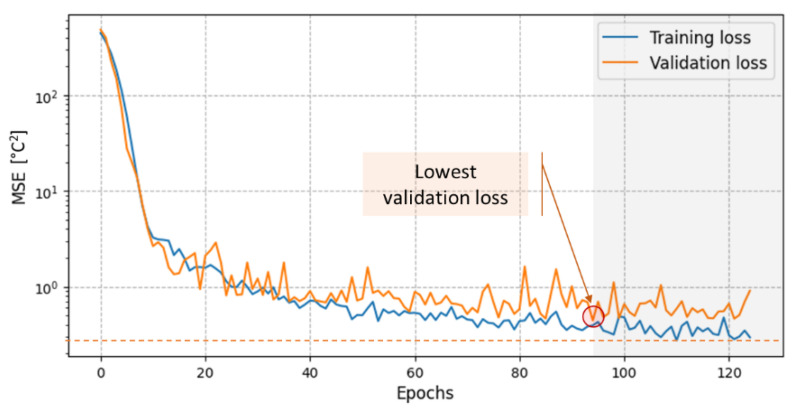
Training and validation loss during training of the CNN, showing characteristic behavior for the chosen architecture (as presented in Figure 3) and the hyperparameters of the training algorithm (125 epochs, batch size = 64, Adam optimizer with lr = 0.001, β_1_ = 0.9, β_2_ = 0.999).

Figure 5 shows the performance of the CNN model on the training and validation datasets during training in terms of mean square error (MSE). Both the training and the validation errors are improving during training, with the most significant improvement to be observable in the first 40 epochs. The training was terminated after the 125th epoch because its training loss went below the uncertainty of the temperature chamber, which is a sign of overfitting. As described in Section 2.4, the model that is stored for testing is the one with the lowest validation loss, which was obtained after the 94th epoch. Its performance on the validation dataset corresponds to an MSE of 0.45 (°C)^2^ or to a root mean square error (RMSE) of 0.67 °C. 

**Figure 6:**
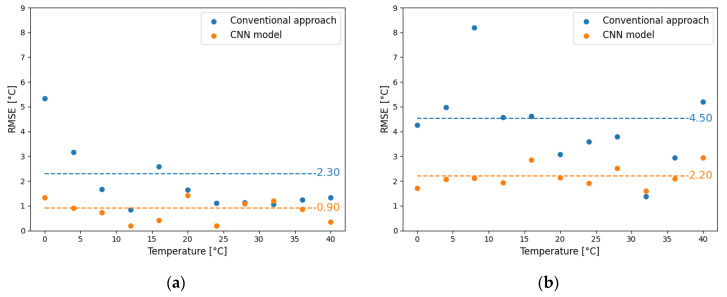
Root mean square error (RMSE) of the conventional (blue) and CNN-based (orange) approach for a 200-m-long fiber section at the beginning of the sensing fiber (**a**) and at the end of the sensing fiber (**b**). The dashed lines correspond to the total RMSE, including all the temperatures.

In Figure 6a,b, we compare the accuracy performance of the conventional method with that of the CNN model that was obtained as described above. The blue dots represent the RMSE of the conventional method at different temperatures, while the orange dots the RMSE of the CNN model. The dashed lines correspond to the total RMSE calculated, including all the temperatures. The RMSE for the 200-m-long section at the beginning of the sensing fiber was improved by 1.4 °C and at the end by 2.3 °C, respectively, by utilizing the CNN approach. The improvement in both cases is significant and shows how well the CNN model can generalize on new data. The errors in temperature estimation at a 30-km sensing distance are much higher than those at the beginning of the fiber. Specifically, the total RMSE of the conventional approach increased by 2.2 °C while that of the CNN model grew by 1.3 °C. These results indicate that both methods have been affected by the decreased SNR at the end of the fiber, but the CNN model shows more tolerance to noise.

The robustness of the CNN model is also demonstrated by the fact that the RMSE varies only slightly with temperature when compared to the conventional method. Especially at 30 km the standard deviation of the errors that are calculated for every temperature is 1.63 and 0.41 for the conventional method and the CNN model, respectively. We also observe that at low temperatures (<16 °C), the RMSE of the LCF algorithm is higher, which is attributed to the low gain and large width of the BGS [34]. 

**Figure 7:**
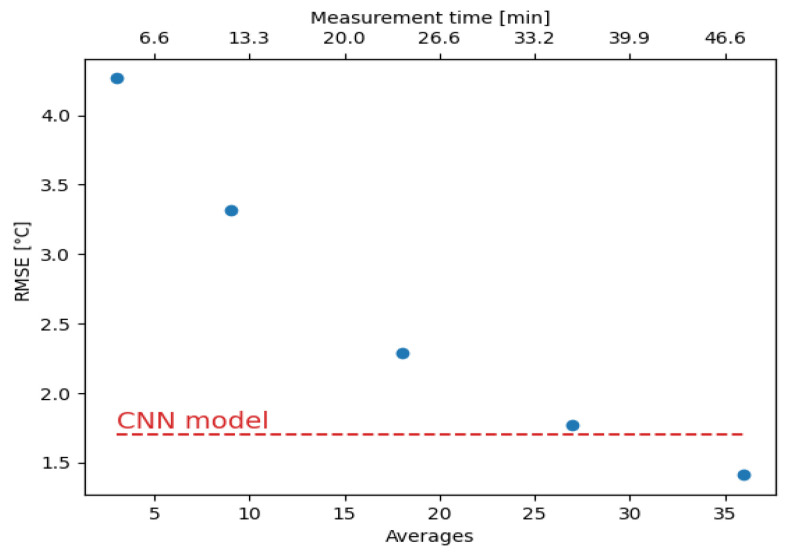
RMSE of the conventional method vs. measurement time and a number of averages at 0 °C.

The conventional method can reach the performance of the CNN model by using data with higher SNR, which can be obtained by increasing the number of averages in the VNA. To estimate the measurement time improvement of the CNN, we conducted additional measurements with a higher number of averages, which resulted in longer measurement times. Specifically, we conducted measurements at 0 °C of the 200-m-long segment at the end of the 30-km-long optical fiber with measurement times up to 48 min. We selected to study our model’s time efficiency at 0 °C because, at this temperature, the RMSE difference of the two approaches is 2.4 °C, which is very close to the difference of the total RMSE (2.3 °C). Figure 7 shows how the RMSE decreases with the number of averages or the measurement time. We observe that the RMSE of the conventional method decreases with the number of averages and is expected to reach a plateau at some point [34]. The conventional method needs measurements of more than 27 averages (36 min) in order to reach the performance of the CNN model, which is evaluated with measurements conducted with only three averages (4 min). In other words, the conventional method requires measurements that are more than nine times longer to outperform the CNN model.

## 4. Discussion

In this paper, we have reported that our proposed CNN-based approach shows great noise-tolerance, and thus it performs very well on data with low SNR. We believe that the performance can be further enhanced if data from the end of the fiber or other positions are also included in the training and validation data sets. This would increase the size and diversity of these datasets. In this work, we chose to keep data collected at the end of the fiber solely for testing in order to show the robustness of the newly proposed method in particular.

We have also shown that owing to the noise tolerance of the CNNs, the measurement time can be decreased significantly. This is of great importance for applications in the field, especially for BOFDA, because the system can only be used for static measurements due to the time invariance requirement that must be met [10]. Unstable environmental conditions during measurements can lead to erroneous estimation of temperature or strain; thus, faster monitoring is less prone to errors due to temperature changes.

In BOFDA, there is no trade-off between spatial resolution and measurement range, and thus it can provide long-range sensing and very high spatial resolution at the same time. Our approach, by reducing the measurement time, can render BOFDA more popular for such applications. 

Future work will investigate the extension of the measurement range in BOFDA using the CNN-based approach. Since low SNR limits the measurement range, we expect that, owing to the noise tolerance, our method shows that the measurement range could be extended above 100 km. We note that the maximum sensing length that is reported in BOFDA is 63 km [35] and 100 km (using additional Raman amplification) [9]. 

## 5. Conclusions

This paper demonstrated a CNN-assisted BOFDA system for time-efficient measurements along with a 30-km-long optical fiber. Our approach overcomes the conventional BOFDA method’s main drawback, which is the time-consuming measurements, performing more than nine times faster. These results can open the way for BOFDA to meet new applications, where the environmental conditions change faster, and thus faster monitoring is required. Potential applications could be in the field of structural health monitoring of large civil infrastructures, like long pipelines and submarine power cables. 

## Data Availability

Not applicable.

## Abbreviations

The following abbreviations are used in this manuscript:

BOFDA	Brillouin frequency domain analysis
BOTDA	Brillouin optical time domain analysis
SNR	signal-to-noise ratio
ANN	artificial neural networks
SVM	support vector machines
BFS	Brillouin frequency shift
LCF	Lorentzian curve fitting
CNN	convolutional neural network
DFB	distributed feedback laser
MZM	Mach–Zehnder modulator
SG	signal generator
FBG	fiber Bragg grating
EDFA	erbium-doped fiber amplifier
VOA	variable optical attenuator
PS	polarization scrambler
VNA	vector network analyzer
FUT	fiber under test
PD	photodiode
iFFT	inverse fast Fourier transformation
BGS	Brillouin gain spectrum
MSE	mean square error
RMSE	root mean square error
